# Factors Affecting Drain Output in Oral Carcinoma

**DOI:** 10.1007/s12663-024-02123-6

**Published:** 2024-02-23

**Authors:** Teertha Shetty, Poonam Joshi, Sanjay Talole, Sudhir Nair, Pankaj Chaturvedi

**Affiliations:** Department of Head and Neck Surgery, Advanced Centre for Treatment, Research and Education in Cancer, Tata Memorial Hospital, Homi Bhabha National Institute, PS Building, ACTREC, Kharghar, Navi Mumbai, Mumbai, Maharashtra 410210 India

**Keywords:** Drain output, Drain volume, Hospitalization, Oral cavity carcinoma

## Abstract

**Introduction:**

Advanced oral carcinoma surgery results in large denuded areas leading to seroma and hematoma. Closed suction drains obliterate dead space and create negative pressure on wound bed. Non-placement or early removal of drain can lead to various complications, while placement for long duration can cause surgical site infection. The study aims to evaluate factors affecting postoperative neck drain volume, guiding surgeons for decision making for time of drain removal.

**Methods:**

The study comprised of 222 patients with oral squamous cell carcinoma who underwent primary tumor resection and neck dissection. Demographical, clinical, and surgical details were retrospectively analyzed.

**Results:**

The mean age of patients was 49.1 years. Majority of patients had advanced disease requiring extensive surgery. Patients with radical neck dissection and those reconstructed with pedicled flap had statistically significant drain volume as compared to those with selective neck dissection and free flaps, respectively. Patients with longer duration of surgery, higher blood loss, low postoperative albumin value, and complications showed increased drain volume. Mean duration of drain removal was 7 days, and all drains were removed by day 10.

**Discussion:**

Advanced stage primary disease, radical and modified neck dissections, PMMC flap reconstruction, longer duration of surgery, and higher blood loss had higher drain output. Thus, patient parameters, tumor factors, and surgery factors influence drain output and hospitalization.

**Conclusion:**

Diligent preoperative and perioperative assessment of various factors can aid trainee surgeons to make decisions for appropriate time for drain removal.

## Introduction

Oral squamous cell carcinoma is common cancer in the Indian subcontinent [1]. They usually present in advanced stages and often necessitate extensive surgeries. For these patients, efforts should be taken to reduce the complication rates in the peri- and postoperative period. Seroma and hematoma formation are common postoperative complications and can lead to wound infection [2, 3]. To reduce these innocuous but potentially deleterious complications, it is a routine practice to use the closed drainage system in the neck wound. The use of drains dates back to Hippocrates where glass tubes, metal tubes and bone were used as passive drains [4–7].

Closed suction drains are placed to create negative pressure on the wound bed and obliterate the dead space. It reduces seroma and hematoma collection in the neck and promotes early wound healing [8]. Conventionally, drain placement in the neck wound is well-accepted and is practiced routinely [9]. However, there are a few contentious issues, which need to be resolved. Early removal of the drain or altogether avoiding the drain can sometimes lead to serious life-threatening complications such as airway obstruction [8]. On the other hand, placement of drains for a long duration can prolong hospitalization costs and can even become a potential route for surgical site infection [10, 11].

Although the decision for drain removal is based on the 24-h volume of drain fluid measured in the postoperative period [12], other factors like type of surgery, consistency of fluid and individual hospital policy also play an important role. Thus, the present study aims to evaluate the factors affecting the postoperative neck drain volume and, thus, guide the surgeon for decision making on the appropriate time of drain removal.

## Materials and method

This is a retrospective analysis of prospectively collected data from October 2019 to March

2020 at the tertiary care center. A total of 222 patients of oral cavity squamous cell carcinoma (OCSCC) were included in the study. A portable, closed, continuous negative pressure drain system was placed in the neck following neck dissection. Those patients without neck dissection or where bilateral neck was addressed, were excluded from the study. This was to achieve uniformity for drain output estimation. Various demographic, clinical and surgical data included the extent of surgical resection, type of reconstruction, extent of neck dissection, intraoperative blood loss, intraoperative and postoperative fluid replacement and the duration of surgery.

The extent of surgical resection was according to the grading followed at the center. The grading is divided into I–VI grades, where grades I and II include minor procedures and procedures under local anesthesia, III and IV include early-staged tumor resection with neck dissection and primary closure of the defect, V and VI include advanced-stage tumor resection with neck dissection requiring locoregional or free flaps reconstruction [13]. The wound-related complications were recorded and classified based on the Clavien–Dindo classification [14]. At Institute, one drain is placed in the anterior triangle of the neck and another in the lateral neck. Drains are removed when the drain volume falls below 25 mL/24 h.

Data were analyzed using the IBM Statistical Package for the Social Sciences software program version 22 for Windows. A *P* value of < 0.05 was considered statistically significant. The results are shown as the medians. Descriptive and univariate analyses were used to identify independent risk factors and determine the association of various parameters about drain.

## Results

The study included 222 patients with 185 males (83.3%) and 37 females (16.7%) with a mean age of 49.1 years (range: 27–82 years). Patients underwent clinical and pre-anesthetic evaluation and were viewed for the pre- and postoperative biochemical values as shown in Table 1.

**Table 1:** Variable showing the drain volume

	Frequency (No.)	Percent (%)	Median drain volume (mL)	*P* value
*Sex*				
Male	185	83.3	495	< 0.05
Female	37	16.7	315	
*BMI*				
Underweight	19	8.6	419	0.45
Normal weight	170	76.6	457.5	
Obese	33	14.8	380	
*Post op albumin*				
< = 3	129	58.1	476	0.58
> 3	93	41.9	425	
*Post Op Hb*				
< = 8	8	3.6	440	0.71
> 8	214	96.4	439.5	
*Tumor stage*				
I & II (early-stage tumor)	88	39.6	369	< 0.05
III & IV (advanced-stage tumor)	134	60.4	482.5	
*Extent of surgery*				
III–IV	64	28.8	353.5	< 0.05
V&VI	158	71.2	498.5	
Nodal status				
Node positive	94	42.3	449	0.89
Node negative	128	57.7	437	
*Neck dissection*				
RND and MND	120	54.1	530	< 0.05
SND	102	45.9	351.5	
*Reconstruction*				
Local flap and primary closure	81	36.5	348	< 0.05
Pedicle flap (PMMC flap)	114	51.3	554	
Free flap	27	12.2	330	
*Duration*				
> 4HRS	187	84.2	495	< 0.05
< 4HRS	35	15.8	329	
*Blood loss*				
< 750 mL	104	46.8	369	< 0.05
> = 750 mL	118	53.2	525	
*Fluid replacement*				
< = 5500 mL	51	23.0	370	0.05
> 5500	171	77.0	476	
*Complications (Clavien–Dindo classification)*				
Grade 1 (any deviation from the normal postoperative course without the need of pharmacological treatment or surgical intervention)	30	13.5	350.25	0.05
Grade 2 (requires pharmacological treatment)	17	7.6	378.54	
Grade 3 (requires surgical, endoscopic or radiological intervention)	22	9.9	398.24	
Death	1	0.5		
Patient without complications	152	68.5	271.5	

*RND* radical neck dissection, *MND* modified neck dissection, *SND* selective neck dissection, *PMMC flap* pectoralis major myocutaneous flap

For statistical analysis, the patients with body mass index (BMI) greater or equal to 25 kg/m^2^ were grouped into a single category and named obese. Most of the patients (76.6%) were categorized as having normal BMI (18.5–24.9 kg/m^2^), 14.8% were obese, and 8.6% were underweight (< 18 kg/m^2^) as shown in Table 1. The drain volume was found to be higher in the group with normal BMI when compared to an obese and underweight category; however, it was not significant (*P* value 0.45). The drain volume in the male patients was more significant (*P* value < 0.05) than that of the female patients as shown in Table 1.

Regarding the biochemical parameters, preoperative albumin was > 3 gm/dL in all patients. However, on postoperative day 2, the albumin value fell below 3 gm/dL in 129 patients (58.1%). However, these patients had higher mean drain volume but were not statistically significant (*P* value 0.58). Similarly, the postoperative hemoglobin did not correlate statistically to the drain output (*P* value 0.71).

Tumors were staged using the American Joint Committee on Cancer (AJCC) 8th edition Tumor Nodal Metastasis (TNM) classification. 15 Majority were stage IV (T4 N0). For statistical analysis, tumor stage 1 and stage 2 were grouped into early-stage cancers and those with tumor stage 3 and stage 4 were grouped into advanced-stage cancers. Advanced-stage cancers had increased drain volume compared to the early-stage disease (*P* value < 0.05). Nine patients underwent radical neck dissection (RND), 102 patients had selective neck dissection (SND), and modified neck dissection (MND) was done in 111 patients. SND had low drain volume (*P* value < 0.05) as compared to the other two groups. There was no difference (*P* value 0.89) in the drain volume between the pathological node-negative and node-positive groups.

Reconstruction of the oral defects was done with various flaps. Primary closure of the defect was done in 39 patients, 42 patients were reconstructed using the local flap, and the rest were reconstructed with locoregional and free flaps as shown in Table 1. Pectoralis major myocutaneous flap (PMMC) was the choice of reconstruction (*n* = 114, 51.3%) in most of our patient due to high volume of low socioeconomic status of the patients and limited availability of the free flap reconstruction cover. The microvascular free tissue reconstruction showed low drain output and was statistically significant (*P* value < 0.05).

Patients with longer duration of surgery (> 4 h) and higher blood loss (> 750 mL) had significantly higher drain output (*P* value  < 0.05). Higher drain output was not found to be associated with a total volume of fluid replaced (*P* value 0.06). Complications were categorized using the Clavien–Dindo classification. Drain output was higher in those with complications (*P* value 0.05). The details are given in Table 1. The mean duration of drain removal was 7 days, and all the drains were removed by day 10.

## Discussion

Surgery for oral carcinoma includes resection of the primary disease and addressing the regional lymph node metastasis by neck dissection procedure. These often result in substantial tissue loss leading to a collection of sizable amounts of blood and exudate under the neck flap [16]. The collection can decrease the perfusion of surrounding tissue and can delay wound healing by preventing the flap apposition. The serous proteinase fluid and blood offer excellent culture media and cause surgical site infection and wound-related complications [17]. The resultant implications are additional interventions, investigations and procedures, increasing the cost of the treatment. The resultant delay can hamper the timely delivery of adjuvant therapy, which is detrimental to the success of the treatment.

Passively acting drain which acts by capillary action or gravitational force is not useful in head and neck surgeries [18]. It fails to drain nondependent areas and frequent dressing to collect the drainage is cumbersome and leads to patient discomfort. Moreover, the drain volume cannot be quantified. The constant soakage at the wound site leads to maceration of the skin and surgical site infection [17–20]. All this disadvantage of an open, passive chain system is overcome by a closed drain system [18]. Thus, the closed drain system is an integral component of postoperative neck management at our Institute. It is placed to create a negative pressure on the wound bed and obliterate the dead space [8].

However, prolonged placement can lead to retrograde infection and limited ambulation [17, 18]. These can be minimized by carefully securing the drain and routine care under aseptic precautions [12]. Even though the use of drains has been debated in the past in our experience, the benefit outweighs the risk. The only predicament is the duration for which it should be kept in situ. The present retrospective analysis was done to assess the various factors that can affect the drain amount in the postoperative period.

In this study, the mean drain output was 371.5 mL and the mean duration of drain removal was 7 days. The female patients were seen to have less drain volume and duration of stay when compared to male patients. However, it should be noted that only 16.7% of patients were females in the study, and also the majority of the female patients presented with less advanced diseases and less extensive surgery, and most were reconstructed with free flaps.

Analyzing BMI, the drain volume was found to be higher in the patients with normal BMI when compared to the underweight and obese patients. Obesity induces adipocyte hypertrophy and hyperplasia, eventually impairing the metabolic functions of the adipocytes, thus storing more fat and overall reducing the dead space [21]. This probably explains the lower drain output as compared to those with normal BMI. Here, as the number of obese and underweight patients was less when compared to those with normal BMI, further evaluation in future studies is needed to conclude it to be an independent factor affecting the drain output.

Low values of postoperative albumin (< 3 gm/dL) had a trend toward higher drain output. This could be attributed to the dilutional hypoalbuminemia due to fluid resuscitation [22, 23]. Proper levels of albumin can help facilitate healing. Inflammation will increase the number of cytokines, or immune system secretions, in the body [24]. This in turn will pull albumin from around the veins and circulate it to the liver in a process that, in a healthy patient, will resolve inflammation and aid in healing. The drain output was found to be significantly related to the tumor stage, extent and duration of the surgery. The extent of neck dissection showed a positive correlation with increased drain output. This can be explained as the neck dissection creates a larger surface of the raw muscle that is denuded of the enveloping fascia, thus creating a larger wound. This can be justified to be the reason for the increased drain output in the radical and modified neck dissection patients when compared to the selective neck dissection patients [16, 17].

Recently, there has been an emerging interest in the role of sentinel lymph node biopsy in head and neck malignancies and robust clinical data support its utility in early oral cancer [25, 26]. The sentinel lymph node biopsy approach, regardless of cost, enables for quick and safe detection of sentinel nodes and has shown potential in guiding selective neck dissection [27–29]. Even though the procedure necessitates surgical experience and specialized equipment, such as pre-operative lymphoscintigraphy and intraoperative gamma probe, there are less neck dissection morbidity and no neck drain placement, resulting in a good quality of life for the patient [30, 31].

Reconstruction of the advanced surgical defect is often complex. The drain volume was similar in patients with local flap reconstruction and primary closure, whereas it was seen to be significantly higher in those with pedicled (PMMC flap) reconstruction. The free flap remains the preferred reconstruction and has the least drain output among all. This observation can be correlated to the size, thickness/bulk of the flap, the vascular pattern of the flap and also the location of the reconstruction where the dead space is created against gravity and for which the drain is required to empty the collection from the dead space [16].

Operating time was found to have a significant correlation with the drain volume and can be explained as long operative time is associated with extensive operations, neck dissection and reconstruction with a pedicle (PMMC flap) flap. As we know, multiple-level procedure is generally associated with increased dissection of tissue and large exposed bony surfaces; it is reasonable to consider that this factor could lead to increased postoperative drain output [32]. The bulky flap (pedicle flap) requires more time to remove the toxic material, blood and serum that collect under the skin flap [17]. Thus, there is the likelihood of collapse of the capillaries and lymphatic channels, thereby increasing the blood flow and increase in lymphatic drainage to this area [17]. Thus, it is shown to have increased high drain output.

We found that the drain volume in patients with postoperative complications was higher than in those without postoperative complications. Post-operative complications are often associated with infection resulting in more inflammation and exudate formation [16]. Thus, patients with higher total drain volume should be carefully monitored for postoperative complications. This study has been shown to have a strong positive correlation between drain volume and intraoperative blood loss. The drain volume was significant (*P* value < 0.05) in the patients who had blood loss of more than 750 mL. Thus, the total intraoperative blood loss can be considered an important factor in predicting the postoperative drain volume. [33, 34]

## Conclusion

This is a descriptive study of the characteristics (volume and duration), clinicopathologic and surgical parameters associated with drains in a large group of patients with oral carcinoma treated surgically. It gives us insight into factors like the advanced stage of the tumor, extensive resection, radical and modified neck dissection, intraoperative higher blood loss, pedicle flap reconstruction and longer duration of surgery that are interrelated and are independent risk factors for increased drain output and subsequently result in longer hospital stays.

However, various interventions can eliminate or reduce the drain output like the sentinel lymph node biopsy in the cN0 neck. Sentinel lymph node biopsy offers less invasive alternative to elective neck dissection and is often brainless. It was reported to be less morbid, thereby reducing the hospital stay [35]. Other interventions can be the pre-operative nutritional build-up to raise the serum albumin levels to reduce the postoperative complications [36], meticulous dissection to reduce the blood loss and reconstruction with free flaps.

Also in this study, we have followed our institutional policy of drain volume of 25 mL/day for drain removal. However, there are studies in the literature where varying amounts of drain output have been considered for drain removal^12^,^34^,^37^. Probably, a strict cutoff could have contributed to a longer hospital stay in our study. A well-designed prospective study is needed to decide on the appropriate cutoff value of drain output for its removal. The patient factors, tumor-related factors and surgical factors can be assessed preoperatively and can aid trainee surgeons and residents in decision making regarding appropriate timing for drain removal and patient discharge.
